# Structural role of the flanking DNA in *mariner* transposon excision

**DOI:** 10.1093/nar/gkv096

**Published:** 2015-02-08

**Authors:** Jacqueline Dornan, Heather Grey, Julia M. Richardson

**Affiliations:** Institute of Structural and Molecular Biology, School of Biological Sciences, University of Edinburgh, The King's Buildings, Max Born Crescent, Edinburgh EH9 3BF, UK

## Abstract

During cut-and-paste *mariner/Tc1* transposition, transposon DNA is cut precisely at its junction with flanking DNA, ensuring the transposon is neither shortened nor lengthened with each transposition event. Each transposon end is flanked by a TpA dinucleotide: the signature target site duplication of *mariner/Tc1* transposition. To establish the role of this sequence in accurate DNA cleavage, we have determined the crystal structure of a *pre*-second strand cleavage *mariner* Mos1 transpososome. The structure reveals the route of an intact DNA strand through the transposase active site before second strand cleavage. The crossed architecture of this *pre*-second strand cleavage paired-end complex supports our proposal that second strand cleavage occurs in *trans*. The conserved mariner transposase WVPHEL and YSPDL motifs position the strand for accurate DNA cleavage. Base-specific recognition of the flanking DNA by conserved amino acids is revealed, defining a new role for the WVPHEL motif in *mariner* transposition and providing a molecular explanation for *in vitro* mutagenesis data. Comparison of the *pre*-TS cleavage and post-cleavage Mos1 transpososomes with structures of Prototype Foamy Virus intasomes suggests a binding mode for target DNA prior to Mos1 transposon integration.

## INTRODUCTION

**Figure 1.**
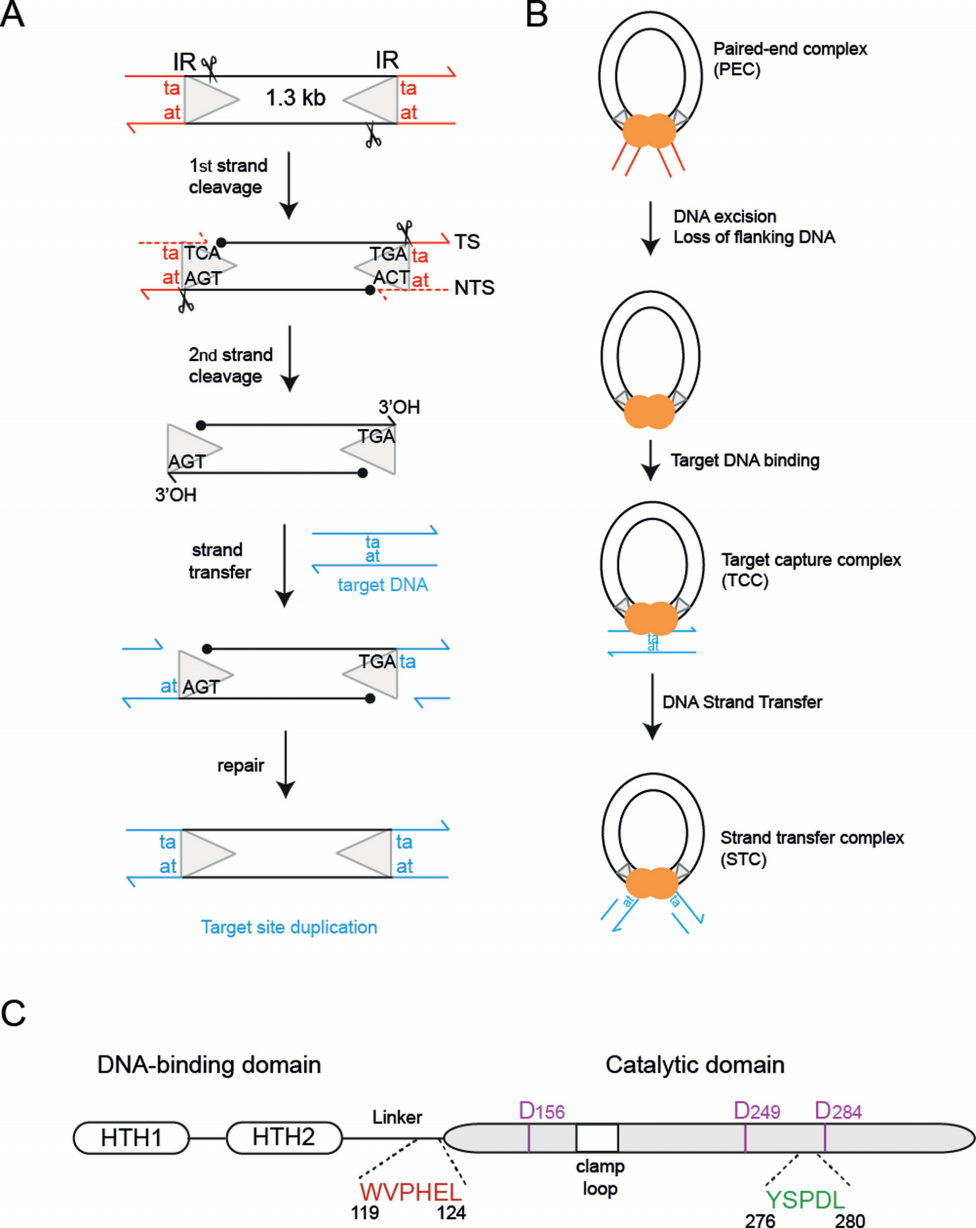
Schematic of cut-and-paste *mariner Mos1* transposition. (**A**) The 1.3 kB Mos1 transposon has a 28 bp IR (triangle) at each end, flanked by the TA target site duplication (red). First and second strand cleavages (depicted by scissors) generate a 5′ phosphate on the NTS (filled black circle) and a 3′OH on the TS (arrow), respectively. Integration of the cleaved transposon into target DNA (blue) duplicates the TA dinucleotide. (**B**) Schematic representation of the nucleoprotein complexes formed during Mos1 transposition. (**C**) Schematic of Mos1 transposase domain architecture, indicating the relative positions of the helix-turn-helix (HTH) domains, the clamp loop and the conserved amino acid motifs.

*Mariner/Tc1* transposons move within and between genomes, shuffling the genetic code and creating genomic diversity or instability. Their mobility in a wide range of species has made them attractive tools for genetic manipulations (1). They move *via* a DNA intermediate by a simple cut-and-paste mechanism, mediated by a transposon encoded transposase (Figure 1A and B). This enzyme cleaves DNA at the inverted repeat (IR) sequence marking each transposon end, then inserts the excised transposon at a TpA site in the new genomic location (Figure 1A). After DNA repair, the TpA target sequence is duplicated immediately adjacent to the transposon ends—a signature of *mariner/Tc1* transposition.

Mariner/Tc1 transposases are sequence-specific nucleases and strand-transferases. The N-terminal DNA-binding domain contains two helix-turn-helix motifs for recognition of a specific transposon IR sequence (2–4). The C-terminal, RNaseH-like catalytic domain contains a DDD/E motif, conserved in other transposases, recombination-activation gene (RAG) recombinases and retroviral integrases (5,6), which coordinates the metals required for catalysis. Mariner transposases contain two additional conserved amino acid motifs: the WVPHEL motif, in the linker between the DNA-binding and catalytic domains; and the YSPDL motif, close to the third aspartic acid of the catalytic triad (Figure 1C).

The eukaryotic mariner transposase Mos1 assembles as a homodimer (7,8) and brings the Mos1 transposon ends together in a paired-end complex (PEC) for excision (9) (Figure 1B). This is analogous to formation of a retroviral intasome, by assembly of an integrase tetramer and viral DNA ends, and subsequent removal of two or three nucleotides from the 3′-viral ends, termed 3′-processing (10). Our Mos1 PEC structure displays a crossed (or *trans*) architecture (4), in common with previously determined transpososome and intasome structures (11–14). In this arrangement the IR DNA bound sequence specifically by the DNA-binding domain of one monomer interacts with the catalytic domain of the other transposase monomer, and *vice versa*. Interactions between amino acids of the linker (containing the conserved WVPHEL motif) and the clamp loop, which extends from the central RNaseH-like catalytic core of the other monomer, stabilize this crossed architecture (4).

The Mos1 PEC structure provides a snapshot of transposition *after* transposon excision by two sequential hydrolysis reactions: the first occurs three bases within the 28 bp IR sequence on the non-transferred strand (NTS) (9); the second takes place exactly at the IR-flanking DNA junction on the transferred strand (TS). The resulting three unpaired bases on the TS are recognized *via* sequence-specific interactions with transposase. These position the TS 3′ hydroxyl moieties close to the catalytic residues for subsequent attack of phosphodiester bonds 5′ to the target DNA sequence (a TpA dinucleotide) and integration at this new genomic location.

TS cleavage must occur precisely at the IR-flanking DNA junction, to ensure the transposon is neither shortened nor lengthened with each transposition event. The T of the TpA target site duplication sequence directly flanking the 3′ end of each IR is critical for accurate TS cleavage in mariner transposition (15,16): mutation of this T to any other base inhibits TS cleavage, whereas mutation of the adjacent 3′ A has little effect on the specificity or kinetics of TS cleavage. The molecular basis for this specificity remains unexplained.

To establish the structural role of flanking DNA in accurate second strand cleavage, we have determined the crystal structure of a *pre*-TS cleavage Mos1 PEC. The structure reveals the route of the intact TS, including the flanking DNA, through the active site before second strand cleavage. The *trans* architecture of this *pre*-TS cleavage complex provides strong support for our proposal that second strand cleavage occurs in *trans*. The structure also reveals a novel role for the conserved mariner transposase WVPHEL and YSPDL motifs in flanking TpA recognition and in positioning the DNA in the active site for precise transposon second strand cleavage.

## MATERIALS AND METHODS

### Transposase mutation, expression and purification

An expression construct encoding Mos1 transposase with the active site mutation D249A was generated by site-directed mutagenesis (Quikchange, Stratagene) of the codon-optimized Mos1 gene (17), according to the manufacturer's protocol. This plasmid also incorporates the T216A mutation allowing soluble expression of Mos1 transposase in *Escherichia coli* (18). The T216A/D249A double mutant transposase was expressed and purified as described previously (18), exchanged into buffer containing 50 mM PIPES pH 7.5, 350 mM NaCl and 10 mM MgCl_2_ and concentrated to 16 mg/ml.

### Strand cleavage and target integration assays

First and second strand cleavage assays were performed as described previously (17). The target integration assay was performed as described previously (19), but using a range of MgCl_2_ concentrations: 10, 25, 50 or 100 mM.

### Preparation of ds DNA substrate

High performance liquid chromatography purified DNA oligonucleotides were obtained from Integrated DNA Technologies and dissolved to 1 mM in TEN buffer (10 mM Tris–HCl pH 8.0, 1 mM EDTA and 100 mM NaCl). The 33 nt TS had the sequence 5′ AAACGACATTTCATACTTGTACACCTGAtagga, with the IR DNA sequence in upper case and the flanking DNA in lower case. The 25 nt NTS had the complementary sequence 5′ GGTGTACAAGTATGAAATGTCGTTT and incorporated a 5′ phosphate, mimicking the NTS cleavage product. Oligonucleotides were annealed in a 1:1 molar ratio by heating to 363 K for 10 min and slowly cooling to room temperature over a period of approximately 2 h.

### Preparation of the *pre*-TS cleavage PEC

The PEC was formed by slow addition of 200 μl of the T216A/D249A mutant transposase (197 μM), in 10 μl aliquots, to 200 μl of the ds DNA substrate (200 μM). The final concentration of the PEC was 43 μM.

### Crystallisation

Crystals were grown by hanging-drop vapour-diffusion in 24-well Linbro plates. The drops contained 2 μl of PEC (43 μM) and 2 μl of well solution comprising 100 mM ammonium acetate, 20 mM magnesium chloride hexahydrate, 50 mM sodium 4-(2-Hydroxylethyl)piperazine-1-ethanesulphonic acid (HEPES) pH 7.0 and 5% (w/v) polyethyleneglycol 8000. The crystals were briefly soaked in a cryo-protectant solution containing 20% (v/v) glycerol and mother liquor prior to cooling in liquid nitrogen for X-ray diffraction experiments.

### X-ray crystal structure determination and refinement

X-ray diffraction data were collected on beam line I03 at the Diamond Light Source to a maximum resolution of 3.09 Å. Crystals displayed C222_1_ symmetry. The X-ray diffraction data were scaled and merged with iMosflm and the statistics are shown in Table 1. Initial phases were determined by molecular replacement, using our structure of the Mos1 PEC with cleaved IR DNA (PDB ID: 3HOS) as the search model in PHASER. The remaining structure was built manually. Restrained refinement was performed with Refmac and Coot and included medium non-crystallographic symmetry restraints on the protein chains. The refinement statistics are shown in Table 1. All structural diagrams were prepared using PyMOL (http://www.pymol.org/) and Adobe Illustrator.

**Table 1.** X-ray diffraction and refinement statistics

Crystal	*Pre*-TS cleavage Mos1 PEC	
PDB ID	4U7B	
Space group	C222(1)	
Cell dimensions	*a* = 96.2 Å, *b* = 339.5 Å, *c* = 160.2 Å	
Wavelength (Å)	0.97625	
Average mosaicity	0.46	
	Overall	Outer shell
Resolution (Å)	92.51–3.09	3.27–3.09
Rmerge	0.134	0.442
Total observations	385605	56422
Unique observations	47937	6890
< *I*>/*σ*<*I*>	8.9	3.4
Completeness (%)	99.7	99.7
Multiplicity	8.0	8.2
Rwork	0.229	
Rfree	0.262	
r.m.s.d. from ideality	0.0051	
Bond Length (Å)		
Bond angle (deg)	0.982	
Chirality (Å)	0.0675	
Ramachandran plot: preferred (%)	94.31	
Allowed (%)	5.59	
Outliers (%)	0.1	
Average *B*-factor (Å^2^)	108.0	
Wilson *B*-factor (Å^2^)	79.6	

## RESULTS

### The T216A/D249A Mos1 transposase double mutant is defective in DNA catalysis

To trap the Mos1 PEC conformation before TS cleavage, we created a catalytically defective Mos1 transposase by mutating the second amino acid residue of the catalytic DDD triad (D249) to alanine. The DDD motif can coordinate two divalent metal ions (Mg^2+^ or Mn^2+^) (8,19). The carboxylate oxygens of D249 and D156 coordinate a Mg^2+^ ion in site 1, whereas D284 and D156 bind the second metal in site 2. We hypothesized that mutating D249 to alanine would preclude binding of a metal ion in both site 1 and site 2, thus preventing DNA cleavage and strand transfer.

**Figure 2.**
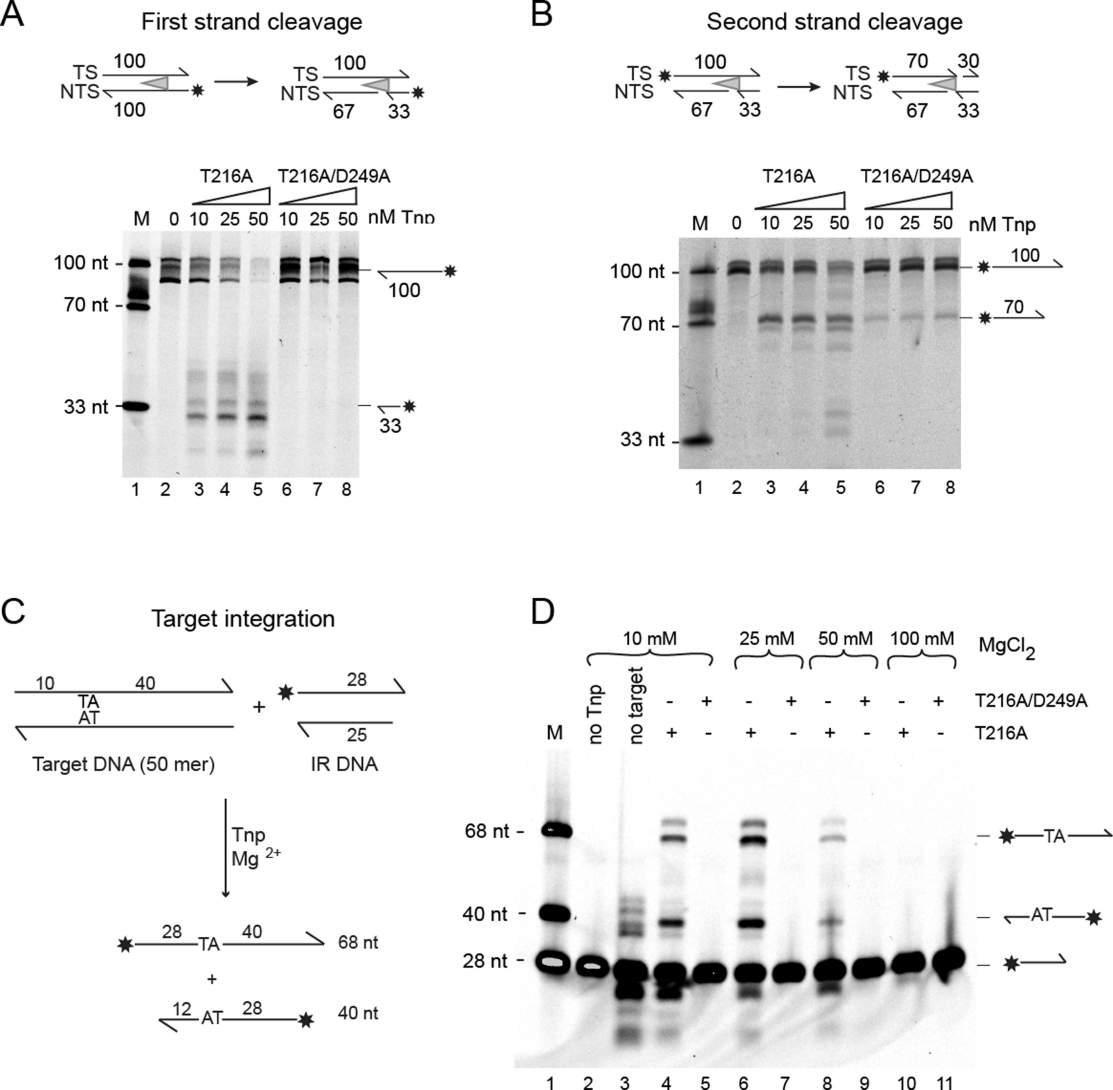
*In vitro* DNA cleavage and integration activities of Mos1 transposase (Tnp) mutants. (**A**) First strand cleavage assay shown schematically (with the asterisk indicating the IRDye® 700 label). Reaction products, separated by denaturing polyacrylamide gelelectrophoresis (PAGE), are indicated to the right of the gel. Lane 1 contains labelled DNA markers and the reaction in lane 2 is without transposase. Reactions in lanes 3–5 were performed with T216A Mos1 transposase, at concentrations of 10, 25 and 50 nM, respectively, whereas reactions in lanes 6–8 contained T216A/D249A transposase. (**B**) Second strand cleavage assay, with conditions identical to those described for the first strand cleavage assay. (**C**) Schematic of the *in vitro* DNA integration assay. Integration of the labelled IR DNA into the top strand of target DNA yields a 68 nt labelled product, whereas integration into the bottom strand produces a 40 nt labelled product. (**D**) Comparison of the activity of the T216A and T216A/D249A Mos1 mutant transposases. Reaction products were separated by denaturing PAGE. Reactions in lanes 4–11 contained transposase and MgCl_2_ at 10, 25, 50 or 100 mM, as indicated. Lane 1 contains DNA markers. The control reactions in lanes 2 and 3 had no transposase or no target DNA added, respectively.

*In vitro* DNA cleavage assays with purified T216A/D249A transposase showed the NTS strand remained intact (Figure 2A) and TS cleavage was strongly reduced (Figure 2B). In an *in vitro* DNA integration assay, using a 50-mer target DNA substrate containing one TpA dinucleotide (Figure 2C), DNA integration by T216A transposase was optimum at 25 mM MgCl_2_. However, no DNA integration was observed using the T216A/D249A transposase at all MgCl_2_ concentrations tested (Figure 2D).

### The DNA substrate for crystallisation of the *pre*-TS cleavage Mos1 PEC

**Figure 3.**
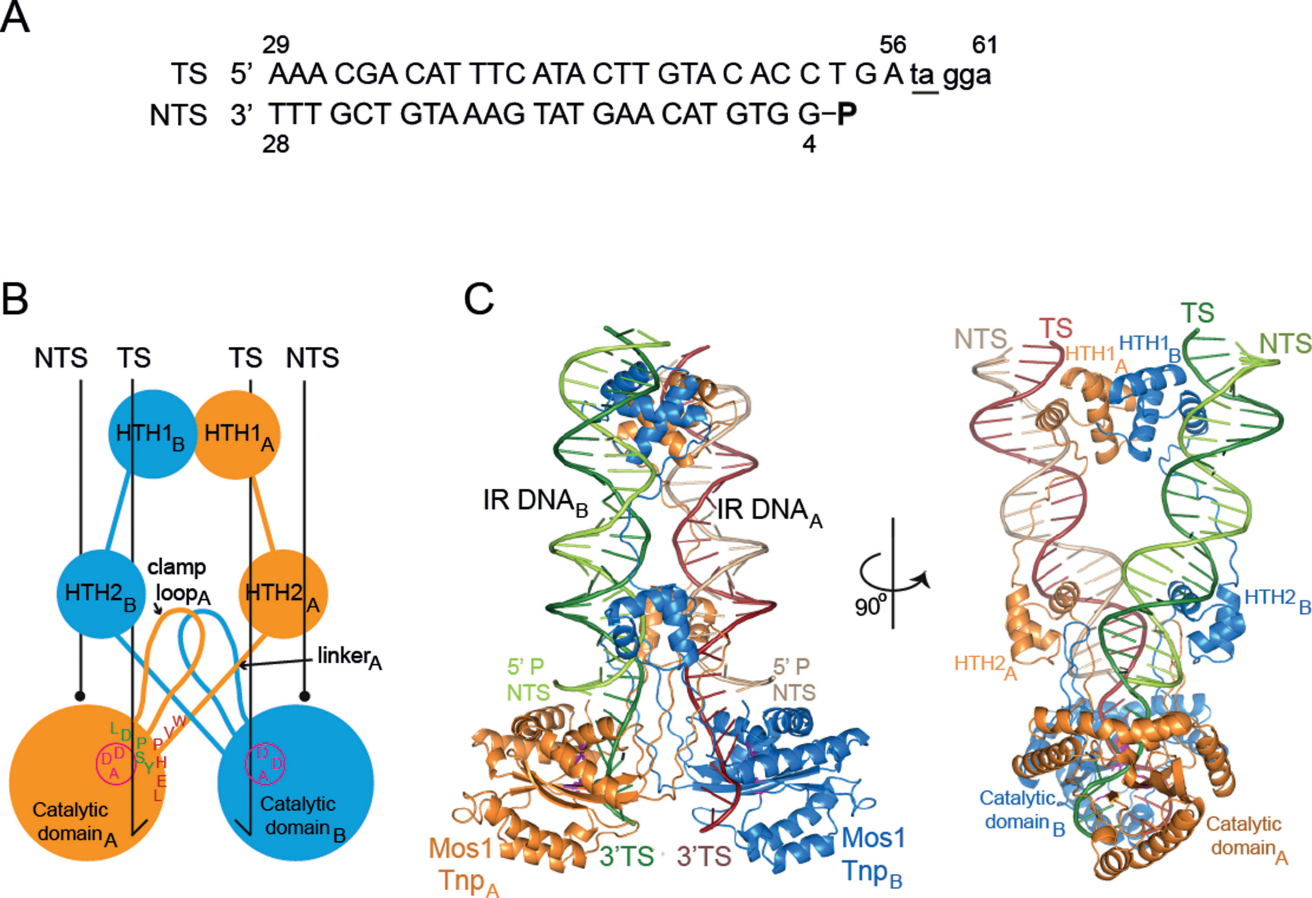
Architecture of the *pre*-TS cleavage Mos1 paired-end complex. (**A**) Sequence of the DNA substrate used in crystallisation of the *pre*-TS cleavage Mos1 PEC, with the flanking TA underlined. The NTS had a 5′ phosphate (indicated by a bold P), mimicking the strand cleavage product. (**B**) Schematic of the *pre*-TS cleavage Mos1 PEC, with conserved amino acid motifs indicated on monomer A (orange). The circle and DAD motif (magenta), indicate the active site of the mutant D249A Mos1 transposase. (**C**) Orthogonal views of the *pre*-TS cleavage PEC crystal structure. Transferred strands are coloured in a darker shade than non-transferred strands.

To prepare the *pre*-TS cleavage Mos1 PEC, we designed a DNA substrate incorporating the IR sequence plus five nucleotides of unpaired TS-flanking DNA, including the TpA dinucleotide abutting the transposon end (Figure 3A). The NTS sequence is the product of first strand cleavage and includes a 5′ phosphate. Previously, we reported that second strand cleavage is enhanced when the DNA substrate has single-stranded flanking DNA compared to base-paired flanking DNA (8), strongly suggesting that the TS-flanking DNA is unpaired in the active sites of the PEC prior to second strand cleavage. Unpairing of the DNA bases flanking the IR sequence could occur during the conformational change that is required between cleavage events, to remove the cleaved NTS from the active site and position the intact TS in the active site for cleavage (8,15).

### Mos1 *pre*-TS cleavage PEC crystals

Equimolar amounts of DNA and T216A/D249A Mos1 transposase were mixed to form the *pre*-TS cleavage Mos1 PEC for crystallisation. Crystals of the *pre*-TS cleavage Mos1 PEC diffracted X-rays to 3.09 Å resolution and exhibited C-centred orthorhombic symmetry (Table 1). Each crystallographic asymmetric unit (ASU) contains one and a half *pre-*TS cleavage PECs (37% of the ASU volume) as shown in Supplementary Figure S1. Two adjacent half PECs, related by 2-fold crystallographic symmetry about the *b*-axis, form one whole PEC.

Crystal lattice contacts between adjacent PEC molecules are predominantly *via* protein–protein interactions. This is distinct from the molecular packing arrangement in our previous, post-cleavage Mos1 PEC crystals (4), which was dominated by additional copies of the IR DNA duplexes interacting with the transposase catalytic domains. Binding of these additional DNA duplexes is precluded in the *pre*-TS cleavage Mos1 PEC crystals, and the flanking DNA is not involved in crystal packing interactions.

### Crossed architecture of the *pre*-TS cleavage Mos1 PEC supports *trans* second strand cleavage

The *pre*-TS cleavage Mos1 PEC structure contains a transposase dimer and two DNA substrates arranged in *trans* (Figure 3B and C). The transposase interactions with IR DNA bases, seen previously in the post-cleavage PEC, are preserved in this complex. Each TS is routed through an active site and the backbone is intact at the IR-flanking DNA junction, consistent with a *pre*-TS cleavage state. As predicted, we do not observe Mg^2+^ ions bound to either site 1 or site 2 in the active site of the catalytically defective T216A/D294A mutant transposase. The *trans* architecture of the complex and the route of each TS through an active site supports our proposal that TS cleavage occurs in *trans* (4).

Three out of the five bases of unpaired TS-flanking DNA are visible in the electron density map (Supplementary Figure S2). T57 and A58 are clearly defined, whereas G59 is less well defined, indicating increasing disorder towards the 3′ end of this strand. There was no density for G60 and A61 which were not modelled.

### Role of the conserved WVPHEL motif in base-specific recognition of flanking DNA

**Figure 4.**
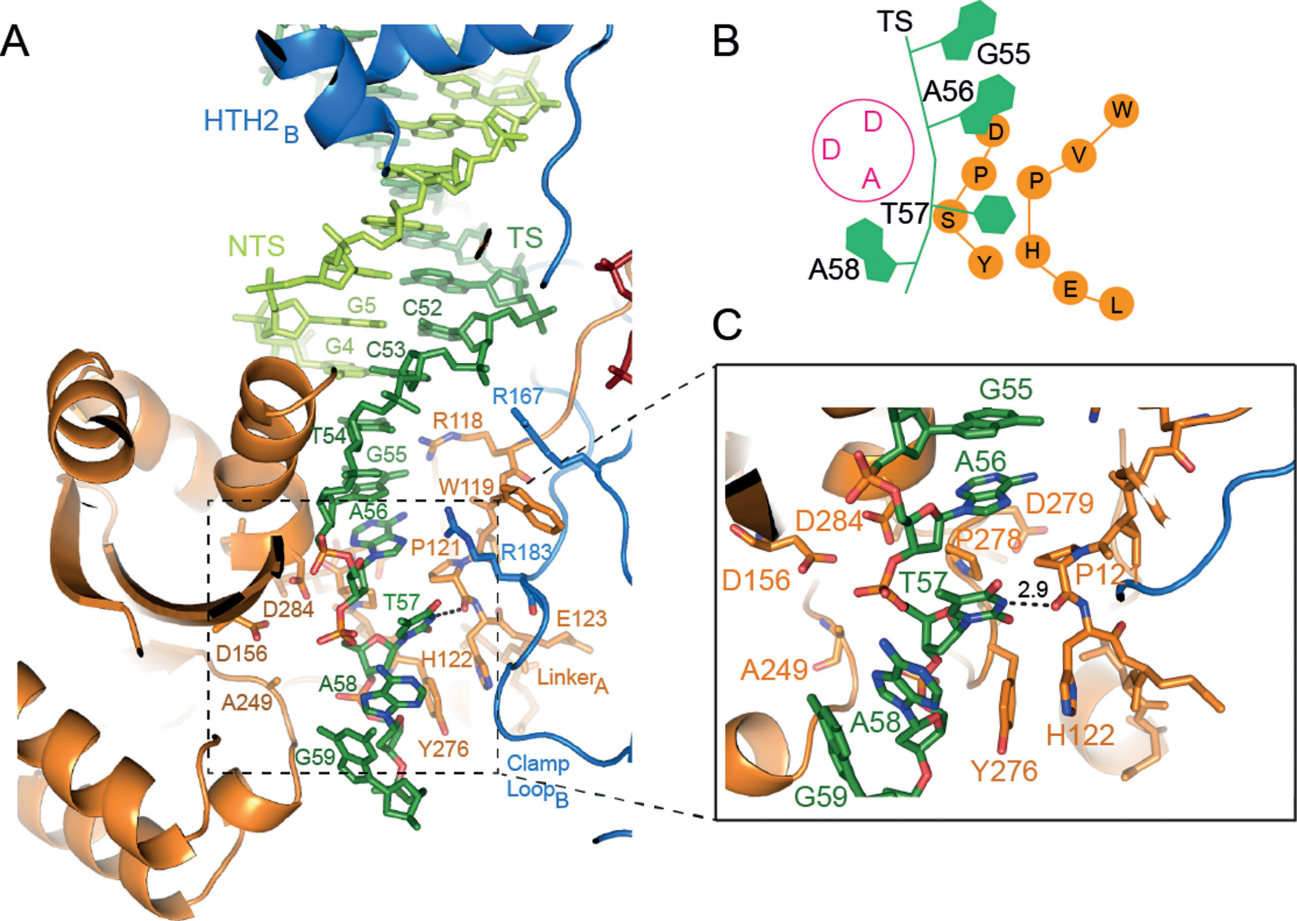
Mos1 transposase interactions with transposon and flanking DNA position the transferred strand for accurate cleavage. (**A**) Expanded view of the catalytic domain of monomer A showing the route of the TS (dark green) and the key interactions between transposase and the TS transposon and flanking DNA (bases T57, A58 and G59). (**B**) Schematic of the TS (green) and conserved transposase WVPHEL and YSPD amino acids motifs (orange) in the vicinity of the DAD mutant active site (magenta). (**C**) Detailed view of the interactions of conserved transposase residues with T57 of flanking DNA.

The TS backbone twists as it passes through the active site (Figure 4A); T57 is de-stacked from adjacent nucleotides with the base flipped into a hydrophobic pocket lined by residues of the conserved WVPHEL and YSPDL motifs. The orthogonal rings of P121 and P278 form a hydrophobic wedge upon which A56 rests (Figure 4B and C). The aromatic rings of H122 and Y276 stack at the base of the pocket, so that only a pyrimidine base is accommodated, while a hydrogen bond between the backbone carbonyl of P121 and the N3H of T57 confers base specificity. By contrast, there are no base-specific interactions with A58, providing a molecular explanation for the report that TS cleavage tolerates any base in this position (16).

### Comparison of the *pre*-TS cleavage and the post-cleavage Mos1 PEC structures

Our previous structure of the post-cleavage Mos1 PEC contains additional copies of the IR DNA duplex which interact non-specifically with the catalytic domains of the transposase. We proposed that these duplexes could occupy the putative binding sites for flanking DNA (4). To compare the flanking DNA in the *pre*-TS cleavage Mos1 PEC with the additional DNA duplexes in our previous post-cleavage Mos1 PEC, we aligned the structures (Supplementary Figure S3A). The equivalent Mos1 protein chains in the *pre*-TS cleavage and post-cleavage PEC structures superposed with an r.m.s.d. of 0.89 Å over 671 C-alpha atoms. The IR DNA positions are also similar in both structures, and the interactions between IR DNA and transposase are preserved. These similarities indicate that the IR DNA and transposase, including the WVPHEL and YSPDL motifs, do not rearrange within the PEC during TS cleavage.

**Figure 5.**
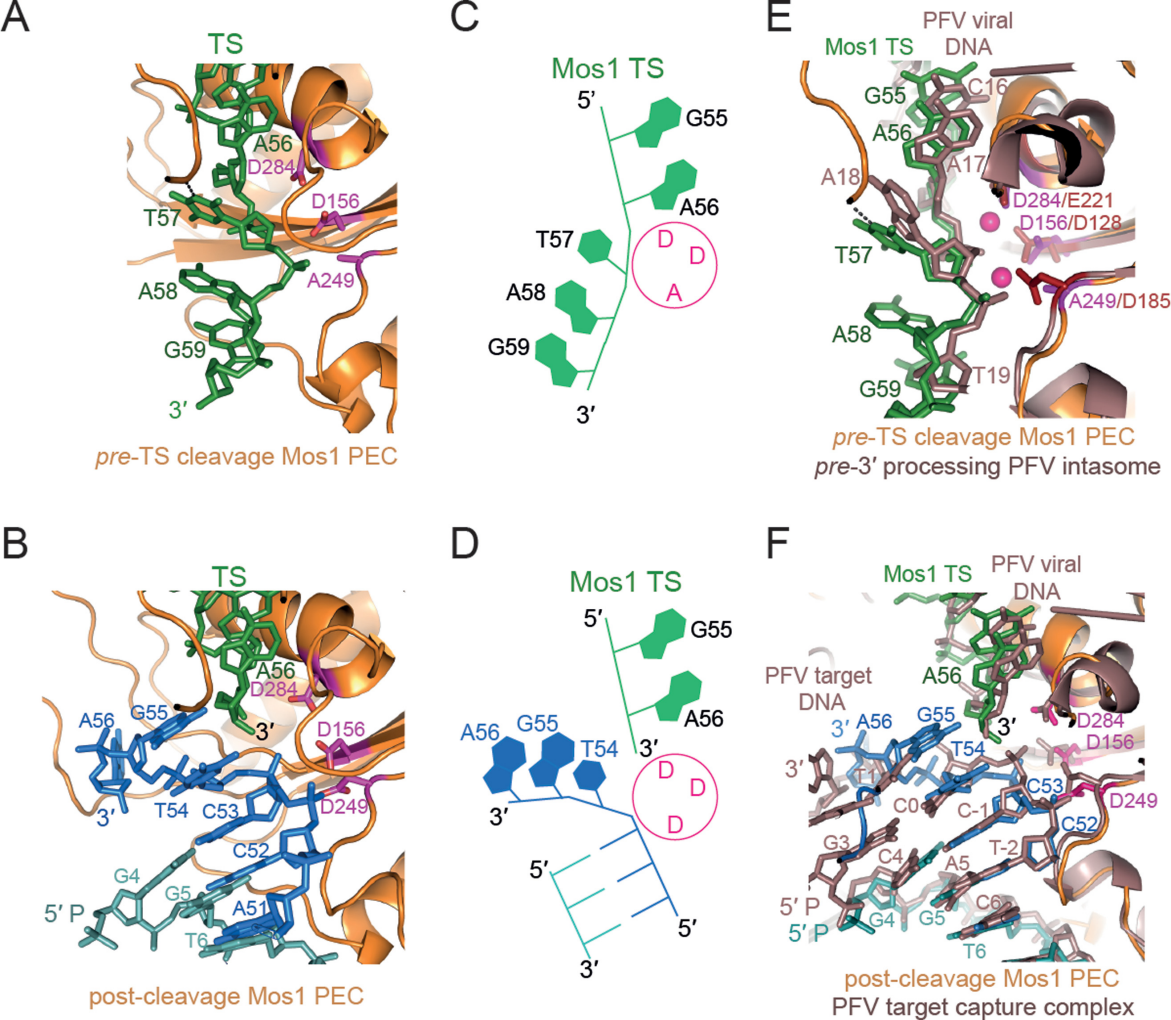
Comparison of the flanking DNA in the *pre*-TS cleavage Mos1 PEC, post-cleavage Mos1 and PFV intasomes. (**A**) Route of the TS (green), including the flanking DNA TpA target site duplication, in the *pre*-TS cleavage Mos1 PEC. (**B**) Position of the cleaved TS (green) and additional DNA duplex (blue) in the post-cleavage Mos1 PEC (PDB ID: 3HOS). The active site residues (DDD) are shown as magenta sticks. (**C**) Schematic of the *pre*-TS cleavage Mos1 PEC structure highlights the 5′ to 3′ polarity of the TS. (**D**) Schematic representation of the post-cleavage Mos1 PEC, showing the 3′ end of the cleaved TS and the opposing polarity of the strands of the additional IR DNA duplex. (**E**) Superposition of the *pre*-TS cleavage Mos1 PEC (orange), where the Mos1 TS is shown in green, with the *pre*-3′ processing Prototype Foamy Virus (PFV) intasome (PDB: E47I), coloured violet brown. (**F**) The post-cleavage Mos1 PEC (PDB ID: 3HOS), shown in the same orientation and colour scheme as part (B), superposed with the PFV target capture complex (PDB ID: 3OS2) coloured violet brown. The additional DNA duplex (blue) in the Mos1 PEC closely aligns with the PFV target DNA.

As we predicted previously (4), the DNA flanking the TS strands in the *pre*-TS cleavage Mos1 PEC (Figure 5A) occupy the same sites as the additional DNA duplexes in the post-cleavage PEC (Figure 5B). However, there are differences in the orientation and polarity of the strands (shown schematically in Figure 5C and D) and in their interactions with transposase (Supplementary Figure S3B). The polarity of the TS-flanking DNA in the *pre*-TS cleavage transposition intermediate is 5′ to 3′, and thus follows correctly the polarity of the TS IR DNA (Figure 5C). The TS-flanking DNA also has the correct TpA sequence directly adjacent to the end of the transposon IR. The orientation of the intact TS in the *pre*-TS cleavage Mos1 PEC closely aligns with that of intact 3′-viral DNA in the analogous *pre*-3′ processing Prototype Foamy Virus (PFV) intasome (20) (Figure 5E).

By contrast, in the post-cleavage Mos1 PEC the polarity of the additional DNA strands in the active sites is 3′ to 5′, opposite to that of the cleaved TS (5′ to 3′), as shown schematically in Figure 5D. Superposition of the post-cleavage Mos1 PEC and the PFV integrase target capture complex (TCC) (21) reveals that the additional DNA in the post-cleavage Mos1 PEC has the same polarity and a remarkably similar orientation as target DNA in the PFV TCC (Figure 5F). These similarities suggest that the additional duplex in the post-cleavage Mos1 PEC represent part of target DNA prior to Mos1 transposon integration. The phosphate linking C53 and T54 of the additional DNA in the Mos1 post-cleavage PEC aligns with the scissile phosphate of the PFV target DNA strand, between C-1 and C0, which is poised in the active site for nucleophilic attack by the 3′-viral end. Thus, T54 of the additional DNA in the Mos1 post-cleavage PEC likely mimics the T within the Mos1 TpA target DNA sequence, into which all *mariner*/Tc1 transposons are integrated (Figure 1). The adjacent nucleotide, G55, may reflect the position of the A of the Mos1 TpA target DNA. G55 makes two base-specific interactions with transposase: the NH_2_ makes a hydrogen bond with the carbonyl oxygen of P184 and N_1_H interacts with the carbonyl oxygen of P121 (4). This hints at a potential additional role for P121 and the WVPHEL motif in target DNA recognition.

## DISCUSSION

The signature TpA target site duplication flanking each end of *mariner/Tc1* transposons is required for accurate and efficient excision of the transposon from flanking DNA. Dissection of the molecular events leading to excision of the *mariner* transposon Hsmar1, revealed that this TpA is required for TS cleavage, the rate-limiting second catalytic step of transposition, but not for the prior and more rapid cleavage of the NTS (15,16). The crystal structure of a *pre*-TS cleavage Mos1 PEC reveals the molecular basis of this requirement. Interactions with the conserved mariner transposase motifs—WVPHEL and YSPDL—play key roles in base-specific recognition of the target site duplication and in orienting the TS-flanking DNA in the active for cleavage precisely at the transposon: flanking DNA junction.

The structural results presented here are consistent with and explain the results of Liu and Chalmers (22), who performed saturating mutagenesis on amino acids of the conserved WVPHEL motif of the related mariner transposase Hsmar1. All amino acid substitutions of the P or the H within the WVPHEL motif produced transposases which were hypoactive in bacterial papillation assays (22). These findings are consistent with the key structural role of these residues in our *pre*-TS cleavage Mos1 PEC structure, assuming that the mutations affect the rate-limiting TS cleavage. Interestingly, mutation of P to a hydrophobic residue (A, C, V or L) or replacement of H with an aromatic residue (F, Y or W) had milder effects than all other mutations (22), implying that most features of the flanking DNA-binding pocket are preserved with these mutations. Substitution of the V within the WVPHEL motif of Hsmar1 transposase, with a G also resulted in hypoactive enzyme (22); our structure suggests that this could be explained by increased flexibility in the motif and subsequent loss of the specific interactions between the backbone carbonyl of P121 and the N3H of T57. The additional observation that alanine mutants of the Y, P and L in the YSPDL motif were hypoactive in bacterial papillation assays (22), further supports our structural finding and highlights the importance of both the WVPHEL and YSPDL motifs in the correct positioning of DNA in the active site for cleavage.

The WVPHEL motif is proposed to fulfil a range of functions during mariner transposition (23). It mediates dimerisation by interacting with the clamp loop in both the *pre*-TS cleavage (Figure 3A) and *post*-cleavage (4) Mos1 PEC structures, thereby stabilising the *trans* architecture of both synaptic complexes. Conservation of these interactions in both the *pre*-TS cleavage and post-cleavage PECs illustrates how this motif facilitates communication between transposase sub-units during transposon excision (16,23). Recently, others have proposed that the WVPHEL motif mediates allostery in mariner transposon end synapsis (22) on the basis that most substitutions of W, V, E or L yielded hyperactive Hsmar1 transposases; alanine mutants of these residues in Himar1 transposase were also hyperactive (24). One explanation is that these mutations relieve mechanisms which down-regulate *mariner* transposition, by disrupting communication between transposase sub-units (22).

Tc1-like transposases contain the consensus sequence motif RKKP in the equivalent linker between the transposase N-terminal DNA-binding domain and the C-terminal catalytic domain (25). A flanking TpA dinucleotide on at least one end is required for transposition of the Tc1-like transposon Sleeping Beauty (26), whereas Tc3 transposition is only moderately reduced if the flanking DNA sequence is mutated (27,28). Therefore, mariner and Tc1-like transposases likely differ in their requirements for and recognition of the flanking TpA dinucleotide during transposition.

The *pre*-TS cleavage Mos1 PEC structure presented here defines a new role for the conserved WVPHEL motif in mariner transposition: base-specific recognition of the thymidine base of the flanking target site duplication and correct positioning of the TS for accurate second strand cleavage. Comparison of the *pre*-TS cleavage and post-cleavage Mos1 PEC structures with the structures of the PFV TCC suggests a route for target DNA in a pre-integration Mos1 complex. Recognition of the target TpA sequence may also involve residues of the WVPHEL motif: mutation of the W within this motif to V in Hsmar1 transposase (22) and the L in Mos1 transposase to S (29) relaxed the target site sequence selection. Structures of Mos1 target DNA integration complexes should shed light on how the target DNA sequence is recognized.

## ACCESSION NUMBERS

The coordinates for the *pre*-TS cleavage Mos1 PEC structure have been deposited in the RCSB protein structure database PDB ID code 4U7B.

## SUPPLEMENTARY DATA

Supplementary Data are available at NAR Online.

SUPPLEMENTARY DATA
