# Supplementary material for: Structural role of the flanking DNA in mariner transposon excision
Source: Nucleic Acids Res. 2015 Feb 8;43(4):2424–32. doi: 10.1093/nar/gkv096 (PMC4344528; doi:10.1093/nar/gkv096)
Supplement: SUPPLEMENTARY DATA [file supp_43_4_2424__index.html]

Structural role of the flanking DNA in mariner transposon excision — Structural role of the flanking DNA in mariner transposon excision — SUPPLEMENTARY DATA 

# Structural role of the flanking DNA in *mariner* transposon excision

## SUPPLEMENTARY DATA

**Files in this Data Supplement:**

- SUPPLEMENTARY DATA
