# Supplementary material for: Structural role of the flanking DNA in mariner transposon excision
Source: Nucleic Acids Res. 2015 Feb 8;43(4):2424–32. doi: 10.1093/nar/gkv096 (PMC4344528; doi:10.1093/nar/gkv096)
Supplement: SUPPLEMENTARY DATA [file supp_gkv096_nar-03573-h-2014-File007.pdf]

Supplementary Information

Supplementary Figure Legends

Supplementary Figure 1. Packing of the *pre*-TS cleavage Mos1 PEC molecules in the crystal. The box indicates the unit cell with dimensions a, b and c as shown.

Supplementary Figure 2. Stereo view of the TS passing through the active site of monomer A in the *pre*-TS cleavage Mos1 PEC. The omit electron density map (contoured at 1.2 σ) is shown as a pale blue mesh. The hydrogen bond between the carbonyl of P121 and the N3H of T57 is shown as a dashed line.

Supplementary Figure 3. Superposition of the *pre*-TS cleavage and post-cleavage Mos1 PEC crystal structures. (A) The *pre*-TS cleavage PEC is shown in colour, using the same scheme as Figure 3C. The TS is green. The post-cleavage Mos1 PEC (PDB ID: 3HOS) is coloured dark grey, except for the additional DNA molecules, previously proposed to represent flanking DNA (labelled FL DNA), which are coloured blue. (B) Close up view of the active sites of transposase monomer A (orange) in the superposed post-cleavage and *pre*-TS cleavage Mos1 PEC structures. The two superposed views corresipond to those shown separately in Figures 5A and 5C, respectively. The TS in the *pre*-TS cleavage structure is shown in green and the cleaved TS in the post-cleavage PEC structure is shown in black.

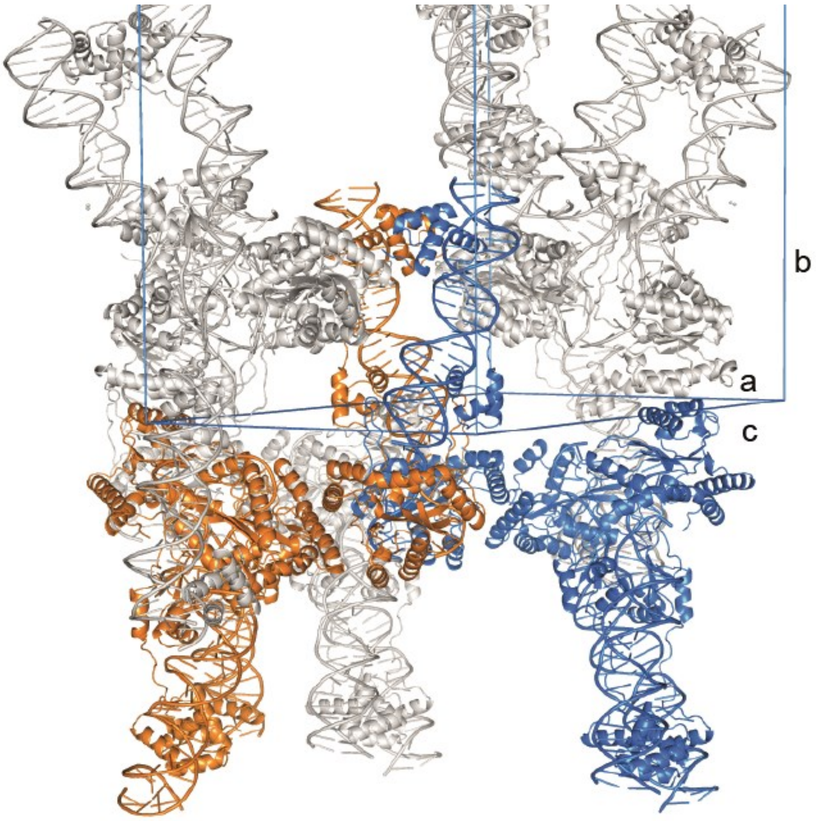

Supplementary Figure 1

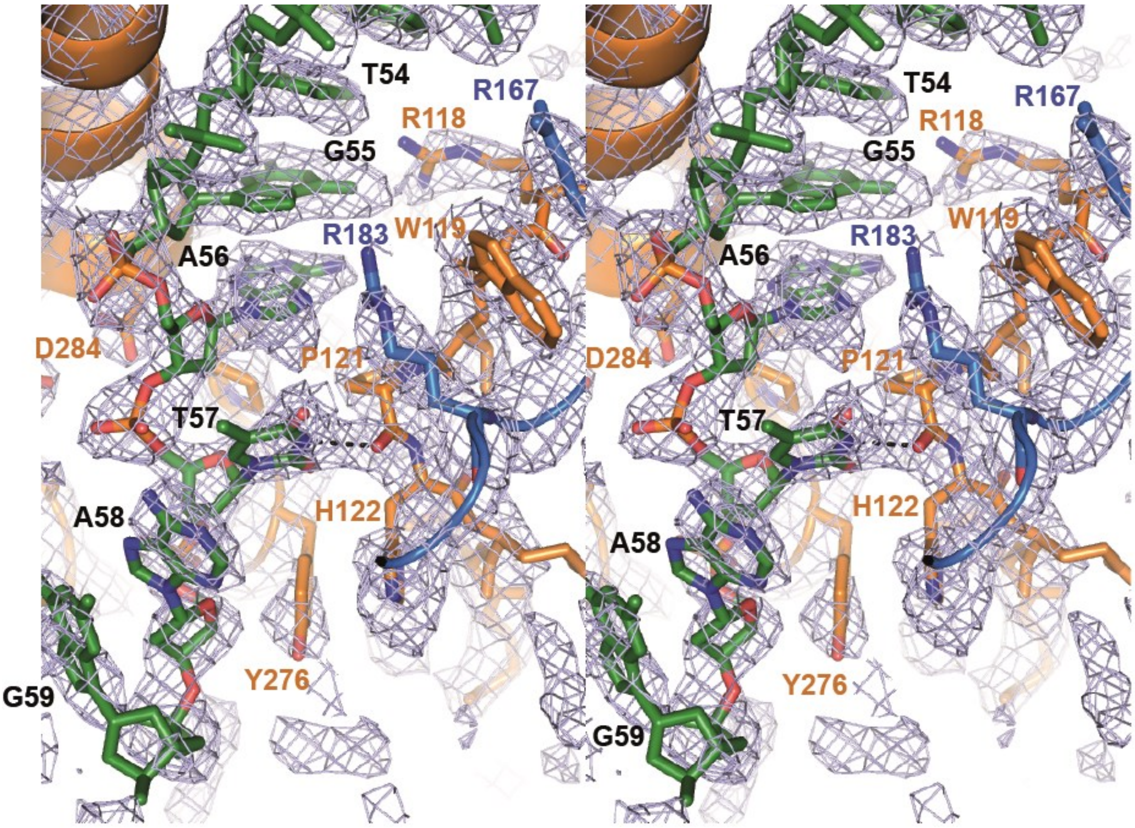

Supplementary Figure 2

A

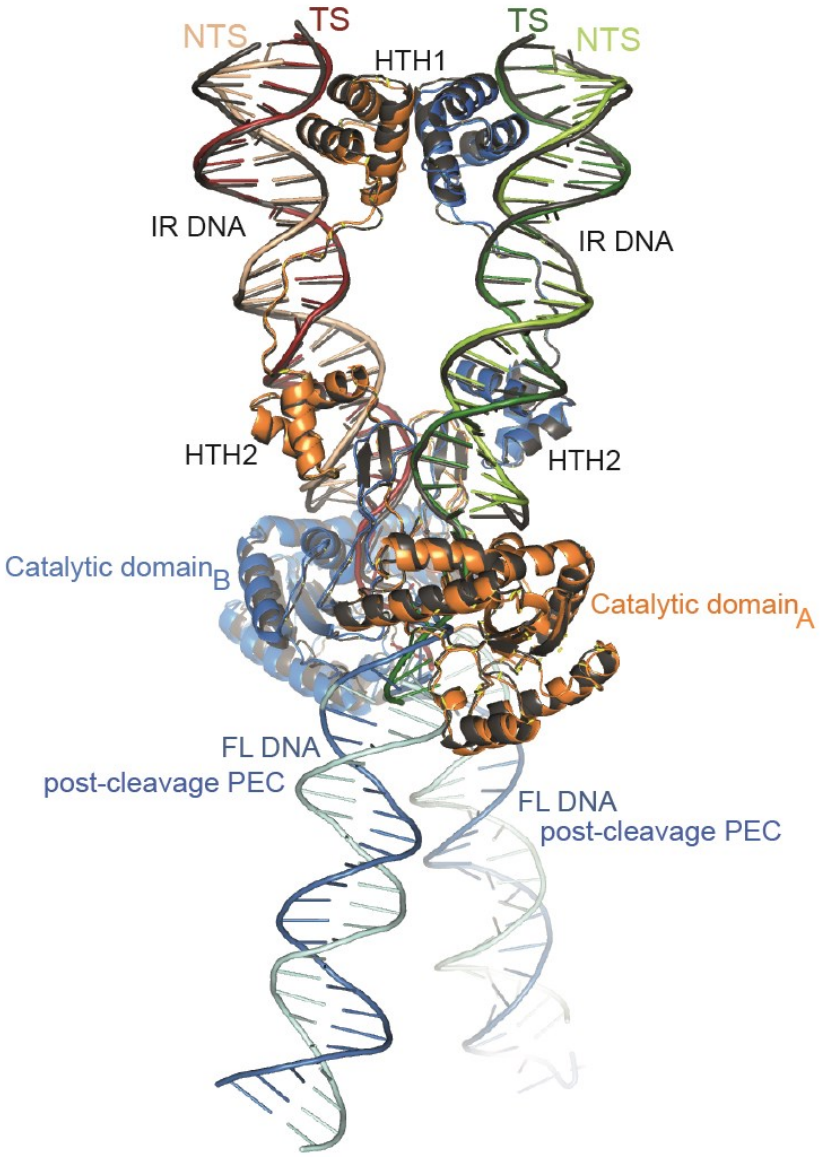

B

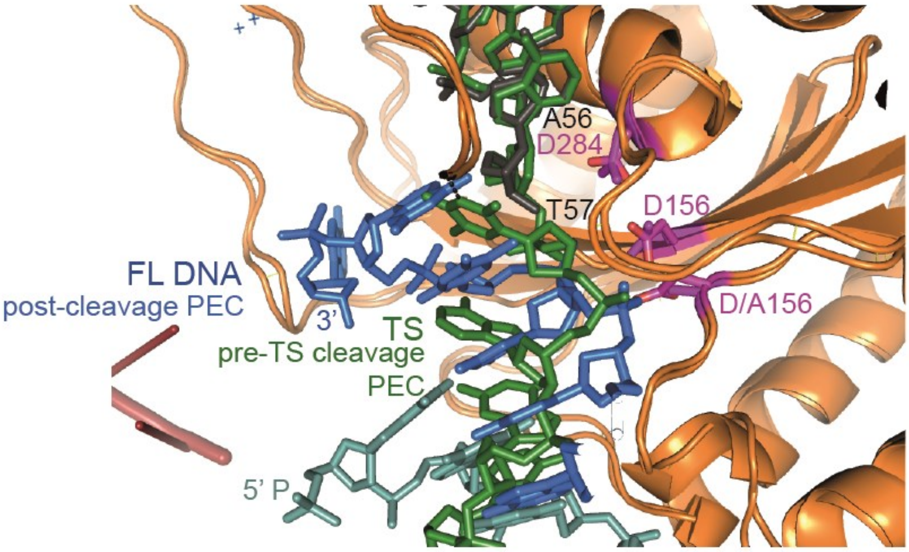

Supplementary Figure 3
